# The Relationship of Gender Roles and Beliefs to Crying in an International Sample

**DOI:** 10.3389/fpsyg.2019.02288

**Published:** 2019-10-10

**Authors:** Leah S. Sharman, Genevieve A. Dingle, Marc Baker, Agneta Fischer, Asmir Gračanin, Igor Kardum, Harry Manley, Kunalan Manokara, Sirirada Pattara-angkoon, Ad J. J. M. Vingerhoets, Eric J. Vanman

**Affiliations:** ^1^School of Psychology, University of Queensland, Brisbane, QLD, Australia; ^2^Department of Psychology, University of Portsmouth, Portsmouth, United Kingdom; ^3^Department of Psychology, University of Amsterdam, Amsterdam, Netherlands; ^4^Department of Psychology, University of Rijeka, Rijeka, Croatia; ^5^Faculty of Psychology, Chulalongkorn University, Bangkok, Thailand; ^6^Department of Medical and Clinical Psychology, Tilburg University, Tilburg, Netherlands

**Keywords:** crying, gender roles, social support, beliefs about crying, emotion regulation

## Abstract

This study aimed to (1) investigate the variation in self ascription to gender roles and attitudes toward gender roles across countries and its associations with crying behaviors, emotion change, and beliefs about crying and (2) understand how the presence of others affects our evaluations of emotion following crying. This was a large international survey design study (*N* = 893) conducted in Australia, Croatia, the Netherlands, Thailand, and the United Kingdom. Analyses revealed that, across countries, gender, self-ascribed gender roles, and gender role attitudes (GRA) were related to behavioral crying responses, but not related to emotion change following crying. How a person evaluates crying, instead, appeared to be highly related to one’s beliefs about the helpfulness of crying, irrespective of gender. Results regarding crying when others were present showed that people are more likely both to cry and to feel that they received help around a person that they know, compared to a stranger. Furthermore, closeness to persons present during crying did not affect whether help was provided. When a crier reported that they were helped, they also tended to report feeling better following crying than those who cried around others but did not receive help. Few cross-country differences emerged, suggesting that a person’s responses to crying are quite consistent among the countries investigated here, with regard to its relationship with a person’s gender role, crying beliefs, and reactions to the presence of others.

The way in which people remember and evaluate their crying experiences are influenced by many factors. These may be whether the cause of crying was positive or negative, how long since the crying occurred (with crying remembered more positively the more time has passed), and social variables, such as the number of people present (Rottenberg et al., 2008a; Bylsma et al., 2011; Vingerhoets, 2013). When researchers consider the social effects of crying, they primarily focus on specific social contexts. However, often neglected in these considerations are other social determinants, such as the socialization of gender roles, which likely play a part in how humans engage in and evaluate their crying experiences.

Although it is well established that women cry more than men (see Vingerhoets, 2013) this difference does not innately appear, with no such differences appearing among infants and small children (see Vingerhoets and Scheirs, 2000). From the age of 11, however, differences in crying proneness and frequency begin to emerge (van Tilburg et al., 2002). Of course, contrary to the notion that women might be biologically inclined to be more emotional, research investigating the role of menarche and crying have found no association with menstrual cycles and crying behaviors (van Tilburg et al., 2003; Romans et al., 2017). Furthermore, much of this change appears to stem from a reduction in boys’ expressions of crying, rather than an increase in girls’. However, this still leaves us with the general finding that women tend to cry more, report more proneness to crying, and often feel better after crying than their male counterparts (De Fruyt, 1997; Vingerhoets and Schiers, 2000; Peter et al., 2001; Becht and Vingerhoets, 2002; van Hemert et al., 2011; Denckla et al., 2014).

## The Role of Culture

Although cross-cultural research has been limited within the crying literature, some research has found a consistent gender difference across at least 35 countries (Becht and Vingerhoets, 2002; Fischer et al., 2004). That is, women cry more frequently and tend to report more positive crying experiences across cultures. This difference is particularly pronounced in many Western countries, with women in those countries showing considerably higher frequencies of crying as compared to women in Asian, South American, and in some West and East African countries (Becht and Vingerhoets, 2002). Despite these gender differences in frequency, however, in some countries, the difference in emotion improvement after crying between men and women was considerably smaller, and in some instances, non-existent, with gender overall explaining very little variance in emotion change.

Further analysis of this research has found that socio-cultural factors may play a role in how crying behaviors are expressed and evaluated (Becht and Vingerhoets, 2002). Indeed, in countries that were more gender equal, wealthier, and where people reported crying often and with little feelings of shame, both men and women tended to report feeling better after crying than people in countries with less equality and wealth. For crying behaviors, the more individualistic a country, the greater the reported crying frequency was for both men and women, with greater gender empowerment related to higher crying frequency among women. However, research by Fischer et al. (2004) alternately found that country level gender empowerment showed no relationship to crying frequency and concluded that crying behavior is more strongly determined by biological rather than social factors.

These results suggest that the influence of culture on gendered differences in crying may play a role in influencing crying behaviors and evaluations. However, research in this area has primarily examined these potential influences using country level indexes and constructs of different socio-cultural factors (e.g., country gender empowerment). They were not able to investigate other possible individual difference factors related to gender and emotion expression. Here, we discuss two related avenues that may help to further explain gender differences found in crying research: (1) individual variation in the acceptance of gender and emotion norms (Frymier et al., 1990; Zahn-Waxler et al., 1996; Fischer and LaFrance, 2015) and (2) individual differences in crying beliefs (Sharman et al., 2018) that may affect engagement in crying and how it is evaluated.

## Gender and Emotion Expression Norms

The widespread distinction between masculinity and femininity in the expression of negative emotions has resulted in a gender-role-consistent pattern of emotion expression, with men tending to express more powerful and hostile emotions such as “anger” and having less tolerance for emotions that display vulnerability, such as sadness and shame (Zeman and Garber, 1996; MacArthur and Shields, 2015). Women, on the other hand, tend to express more vulnerable emotions, such as sadness, fear, and shame, rather than anger (Fischer and Manstead, 2000; Fischer and LaFrance, 2015). Indeed, early parental socialization of emotion expressions in children appears to encourage more expression of anger and less expression of sadness in boys, whereas the opposite is true for girls, with parental socialization resulting in more displays of sadness and less anger than boys from as early as pre-school (Fivush et al., 2000; Brody, 2001; Chaplin et al., 2005). Furthermore, there is an important influence of peers on emotion expression, whereby children are more likely to emphasize gender-role-consistent emotion behaviors when interacting with their peers (Zeman and Garber, 1996; Chaplin and Aldao, 2013). In cultures where gender differentiation for emotion expression is pronounced, this socialization tends to culminate in the attitude that showing vulnerable emotions is a weakness for boys, illustrated with the well-known saying that “boys don’t cry” (MacArthur and Shields, 2015).

Given this socialization, perhaps unsurprisingly, differences in shame felt when crying have been found, with men reporting greater shame than women across the countries studied (Becht and Vingerhoets, 2002). However, this effect was small, and indeed, gender differences, particularly regarding mood and emotion ratings of crying across some of crying research have shown small or no effects (e.g., Williams, 1982; Lombardo et al., 1983; Peter et al., 2001). Other research has alternatively focused on individual differences in beliefs about social roles. For example, Ross and Mirowsky (1984), employing a US sample, focused on men’s crying to understand if sex-role orientation showed an association with high or low crying expressions, with the authors hypothesizing that non-traditional men were more likely to reject stereotypical masculinity. Analyses revealed a relationship between the strength of men’s sex-role orientation and their crying frequency, such that men who adhered to more traditional sex-roles reported lower crying frequencies in response to sadness than non-traditional men. These results have led to suggestions that the differences found between men and women in crying research might be better explained by a person’s perception of gender role patterns (Vingerhoets et al., 2000). That is, the extent to which a person endorses attitudes and behaves consistently with their gender role may influence both their behavior and emotion evaluations of crying. Despite these claims, however, there is a dearth of research on gender roles and their influence on crying behaviors and evaluations. Although there are clear gendered differences in reported crying behaviors and emotional evaluations following crying, understanding the influence of individual differences in gender roles may help researchers to understand some of the variability in crying experiences reported. Moreover, the measurement of crying behaviors has often been limited to crying frequency, which provides an estimate of recent crying but does not provide more detailed information regarding a single crying experience.

## Crying Beliefs

Crying research has tended to focus on how people evaluate their crying experiences by measuring whether people feel better or worse following crying (see Becht and Vingerhoets, 2002; Bylsma et al., 2011). Alongside monitoring changes to their own internal mood and emotional states across time, people also appear to use broader evaluative strategies to understand their crying experiences through beliefs that they hold about how crying works generally and in different social contexts (Sharman et al., 2018).

Crying beliefs likely develop through a combination of prior experience and social expectations regarding crying. Indeed, general beliefs about the benefits of crying have changed little over the last 150 years with crying in the media almost consistently promoted as beneficial and, if suppressed, harmful to wellbeing (Cornelius, 2001). Yet, despite this overall saturation in the media, women are still more likely to endorse beliefs that crying is positive. Certainly, there is some evidence that beliefs about the effect of crying show small gender differences, with women believing that crying is more helpful than do men (Sharman et al., 2018). Although the research on crying beliefs is limited, these positive beliefs might help to explain why women rate feeling better than men following crying, where expectations and beliefs about an emotional experience might help to direct that emotional experience (Bastian et al., 2012). However, these gender differences were not always consistent, with beliefs that crying was unhelpful in private or social situations showing no gendered differences (Sharman et al., 2018). Given that men are more likely to be socialized to believe that crying is a weakness, particularly in social situations, we would expect beliefs to show more of a gendered effect. These results suggest that beliefs about crying may not be as salient to gender stereotypes and because of this, these beliefs may actually mediate the impact of gendered emotion norms surrounding crying.

The current study investigated the individual variations in gender roles across genders and its links with crying behaviors, emotion change, and beliefs about crying. Our primary aim was to understand the function of gender roles and socialization in crying beliefs, crying intensity, and change to emotion following crying, regardless of country. Importantly, in this research, we have referred to emotion, rather than mood as others have done (e.g., Becht and Vingerhoets, 2002). Specifically, we were interested in negative emotions of relatively short duration that caused crying and how those emotion states may have changed immediately following crying, rather than moods, which are generally considered to be longer lasting (Rosenberg, 1998). Furthermore, we also distinguish between GRA and self-ascription to gender roles. As crying behaviors, beliefs, and emotional evaluations likely encompass an interplay of both attitudes toward gender roles (e.g., “crying is for girls”) and degrees to which someone personally subscribes to those gender roles, we include both measures in this research.

We hypothesized that there would be relationships between reported gender, GRA, self-ascribed gender roles, beliefs that crying is helpful, change in emotion following crying, and crying intensity. Such that (H1a) women would be more likely to endorse more feminine gender roles, with men more likely to endorse more masculine gender roles. Furthermore, (H1b) participants who endorsed more masculine gender roles would report less intense crying, (H1c) worse emotions following crying, and (H1d) beliefs that crying is less helpful overall. Conversely, participants who endorsed more feminine gender roles would display higher crying intensity, improved emotion ratings following crying, and beliefs that crying is more helpful overall.

**Figure 1:**
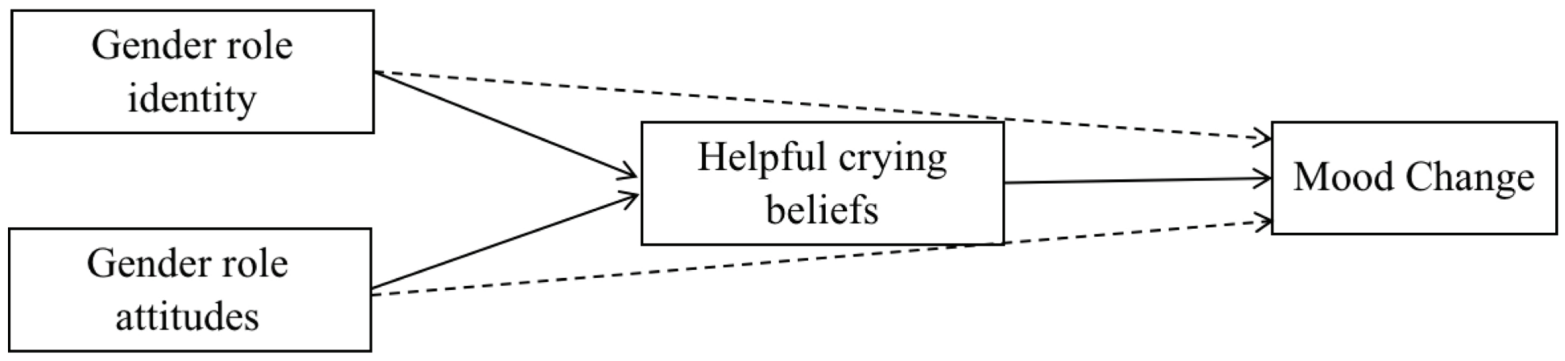
Model of predicted pathways between gender roles, beliefs about crying (BACS), and crying intensity.

We hypothesized that (H2) the relationship between gender roles and perceived emotional change following crying would be accounted for by beliefs that crying is helpful. However, we expected that effect to be observed when recency of the crying episode was accounted for, as crying is more likely to be remembered as generally more beneficial the more time that has passed since a crying episode (Vingerhoets, 2013). Thus, a stronger endorsement of feminine gender roles would be related to more beliefs that crying is helpful, leading to more positive emotion ratings following crying. We similarly hypothesized (H3) that the relationship between gender roles and crying behavior (i.e., intensity of crying) would be mediated by beliefs that crying is helpful. Specifically, participants who subscribed to more feminine gender were expected to report beliefs that crying is helpful, leading to greater crying intensity (i.e., time spent crying and the strength of tearing during the last episode^1^; see Figure 1). Notably, despite general cross-country differences, we believed that gender roles and their respective relationships with crying would remain consistent across countries. To explore whether these relationships were sensitive to country differences, mediation models (H2 and H3) were analyzed to check if this effect was robust across countries, or whether these relationships differed depending on country.

Although the primary aims of this research addressed the potential influence of gender roles in crying behaviors and emotion change following crying, we were also interested in the extent to which the social context might influence people’s evaluations of emotion change following crying. That is, what role does the presence of others play in determining whether a crying person feels better, worse, or experiences no change after crying?

## Crying and the Social Context

Evolutionary explanations of crying have suggested that crying mainly serves interpersonal purposes, such as soliciting help and facilitating social bonds (Gračanin et al., 2018). Crying research has, thus far, shown convincing evidence for this view, finding that shedding tears in the presence of others appears functional. Specifically, the evidence indicates that crying is likely a social signal that encourages more helping behaviors, sympathy, succor, and less avoidance from nearby others (Hendriks and Vingerhoets, 2006; Hendriks et al., 2008; Provine et al., 2009). However, overwhelmingly the methodology used in this research has involved participants rating their willingness to help when presented with vignettes or images of crying (or sad) faces, with little exploration from the perspective of the crier. Moreover, the available evidence that crying encourages helping behaviors in others comes from experimental studies conducted in primarily Western contexts.

Some research focused on the crier’s experience has begun to emerge, with research comparing people who had lost the ability to cry and those who could. For example, Hesdorffer et al. (2017) found no differences between the two groups on their ratings of well-being but did find that people who could cry felt more socially connected with others than those who could not. These findings suggest that crying may assist in creating feelings of social connectedness, yet it is still unclear whether that social connection and help happens during the crying experience itself or, perhaps, because crying encourages a person to seek help afterward. Although study participants express willingness to help when shown images of people crying more than when shown images of the same people without tears (Hendriks et al., 2008; Provine et al., 2009), overall, there has been little research into whether help is actually received by criers. Specifically, it is unclear whether this help is actually functional in improving criers’ emotional state, and furthermore, whether help is received similarly in different cultural contexts.

Crying is more likely to occur in the presence of family and friends than strangers (Vingerhoets et al., 1997, 2000; Nelson, 2005). Yet, it is unclear whether crying among familiar others is more beneficial in terms of help provided and improved emotional state following crying. Of course, it is also possible that someone may provide help to a crying person, but that the crier perceives it to be unhelpful. Thus far, research is scant on whether social crying is beneficial from the perspective of the crier. However, Bylsma et al. (2011) investigated mood change and its relationship to the number of people present. They found that having another person present was associated with an improved mood, whereas crying alone or with more than one person was associated with worse emotion states following crying. A secondary analysis of the same data set, but instead measuring cathartic crying (i.e., a feeling of emotional release following crying) found that feelings of catharsis were related to socially supportive responses (Bylsma et al., 2008). However, this research did not distinguish between help provided from strangers or persons known to a crier and whether there are cultural differences in how social support is interpreted by the crier.

Therefore, a secondary aim of this research was to understand if the presence of others when a person is crying influences how helped or supported they feel when they cry and whether this influences how emotion is evaluated following crying. We hypothesized that: (H4) similar to previous research findings, the crier’s emotional state would improve most when crying with one other person present. Specifically, when compared to two or more people present when crying, the crier’s emotional state would improve most when one person is reported as present; (H5) when the person is known to the crier, criers would be more likely to report that help was provided; (H6) when a crier reports that they have been helped, they would be more likely to report feeling better following crying compared to when no help is reported; (H7) those who believe that crying in social contexts is unhelpful would be less likely to show an improved emotional state when crying in the presence of others compared to when alone. Specifically, we predicted that those who endorse more beliefs that crying is unhelpful in social contexts would rate a worse emotional state following crying when one or more person is present during crying compared to those crying alone. Given the lack of research across cultures in this area, we also explored possible cross-country differences within these hypotheses.

All hypotheses and analyses were pre-registered before data collection and can be found on the Open Science Framework (OSF) at https://osf.io/y37xz/. All materials, data files, and analyses can be found on the OSF at https://osf.io/xvdkz/.

## Materials and Methods

### Exclusion Criteria

Participants were included in the study if they were aged between 18 and 40 years, stated that they resided in one of the target countries (see below), and could remember a previous crying experience that was caused by a negative emotional event, which was not caused by something they read in a book or watched on television or in a movie. These criteria were chosen to ensure that participants were comparable across countries in terms of age and crying experiences (i.e., due to a personal negative event). Eighty-three participants did not meet these criteria. Specifically, they indicated that they resided outside of one of the specified countries, their age was outside 18–40 years, they described their last crying experience as caused by watching a film or video clip rather than a personal experience, or stated they could not remember their last crying event at all.

### Participants

Following exclusion, a total of 893 participants completed the survey, comprising 508 women and 379 men, and a further 5 people, 4 who identified their gender as “other” with some specifying as non-binary or gender queer, and 1 person who did not answer this question (*M*_age_ = 22.81 years, SD_age_ = 5.28). Given the small number of participants who identified their gender as “other,” we did not include these participants among analyses that involved gender. However, they were included in all other analyses.

Participants were recruited in Australia, Croatia, the Netherlands, Thailand, and the United Kingdom. These countries were chosen because they were the home countries of the authors, who shared a mutual interest in crying research. Table 1 provides an overview of participant characteristics from each country. All participants were screened *via* their place of residence and aged between 18 and 40 years, and for all countries, the most common response for the question asking how long they have lived in that country was that they were born and raised there. Ethics approval for data collection across all countries was granted *via* the University of Queensland and the National Statement on Ethical Conduct in Human Research, and the University of Amsterdam ethics committees. Participants were collected *via* student samples at each collaborator’s university, collected from the broader community based on their place of residence, or from an online sample (*Prolific*; www.prolific.ac). Participants were compensated through course credit at each respective university, and online volunteers were compensated £10 per hour for their participation.

**Table 1:** Participant demographics for each country, including number of participants by gender, mean (SD) age in years, and the percentage of participants who indicated they were born and raised in that country.

Country	Men	Women	Non-binary/not specified	Age	% born and raised in that country
Australia	78	94	3	22.83 (4.39)	56
Croatia	75	79	1	23.59 (6.36)	91
Netherlands	75	150	0	21.05 (4.02)	90.2
Thailand	75	88	2	24.72 (4.88)	94.5
United Kingdom	76	97	1	22.53 (6.07)	80.5

*For participants who indicated they resided in Australia, the second most common response for the length of time lived in Australia after ‘born and raised here’ was 11–15 years*.

### Measures

#### Triggers

To ensure participants met the criteria for the study, they were asked if the last time they cried was primarily out of sadness, frustration/powerlessness, anger, or something else with an option for free text entry. Participants were also asked the reason they were crying by selecting if they were crying because of something that happened to you, because of something that happened to someone else (e.g., seeing someone hurt in real life), or “other” with an option for free text entry. Participants who stated that their reason for crying was due to a trigger that was not caused by something either personal to them (e.g., watching a movie), or because of a positive experience were excluded from analysis.

#### Emotion Change

Emotion change was measured on a single item asking how participants felt immediately following crying compared to before on a 7-point scale (1 = much better and 7 = much worse).

#### Crying Intensity

Intensity of crying was measured using a standardized composite score of two items measuring the length of crying time and amount of tearing during the last crying episode. These items were “how long did you cry for?” measured from 1 (less than 5 min) to 5 [continuous (starting and stopping) over a long period of time] and “how intensely did you cry?” rated from 1 (tears in eyes) to 5 (tears down face with vocal sobbing and body movements). These two items were significantly correlated (*r_s_* = 0.39, *p* < 0.001) and combined using standardized unit weights.

#### Crying Frequency and Time Passed

Participants were asked to indicate how long it had been since they last cried. This was measured categorically on a 6-item scale (1 = in the last day, 3 = in the last month, and 6 = more than 6 months ago). Participants were also asked to estimate the number of times they cried over the last month for any reason including crying for positive or negative reasons. Importantly, where participants provided a range of crying frequency (e.g., 10–15 times), an average of their estimate was imputed, or the minimum crying experience identified, for example “more than 10 times”, was imputed as “11” times in the last month. This occurred for 65 participants who gave a range or estimate of their crying frequency. If no clear frequency was identified in a response, such as “a lot,” then the field was left empty.

#### Social Context

Participants were asked how many people they were with measured on a 5-item scale (1 = alone, 2 = one other person, 3 = two other people, 4 = three other people, and 5 = four or more other people). To measure if support was received from a person they were with, participants were asked “If you were with one or more people, did you receive support (e.g., emotional, informational, or active help) from them?”. Support was measured on a 4-item scale (1 = yes, 2 = unsure, 3 = no, and 4 = not applicable). To understand if the person they were with was known to them, participants were asked “If you were with one or more people, did you know them or were they a stranger?”. Responses were recorded across four items, 1 = stranger, 2 = acquaintance, 3 = both stranger(s) and acquaintance (s), or 4 = close friend or family member.

#### Beliefs About Crying Scale

The Beliefs About Crying Scale (BACS) consists of three subscales, comprising a total of 14 items (Sharman et al., 2018). Each item is measured on a 5-point scale (1 = Never, 5 = Always), with three subscales, Helpful (seven items; *α* = 0.73; e.g., “after crying I feel an emotional release”), Unhelpful-Individual (three items; *α* = 0.65; e.g., “Crying makes me feel worse when I’m alone”), and Unhelpful-Social (four items; *α* = 0.78; e.g., “it’s embarrassing when I cry around friends or family”). Given the large sample, small number of items, and lack of hypotheses for Unhelpful-Individual scale, we considered the lower reliability score acceptable for the purposes of this research. Higher scores on the Helpful subscale indicate greater belief in crying as helpful for wellbeing and emotional recovery. Higher scores on the Unhelpful-Individual scale indicate more beliefs that crying leads to feeling worse when alone. Higher scores on the Unhelpful-Social scale indicate more beliefs that crying is unhelpful in social settings because it can lead to feelings of shame and embarrassment.

#### Gender Roles

Self-ascribed masculinity-femininity was measured using the Traditional Masculinity Femininity Scale (TMF; Kachel et al., 2016). This scale measures gender role identity (facets of self-subscribed masculinity-femininity) with items that do not depend on culture and time, as they rely on a person’s own definition of masculine/feminine that would be shaped by their culture and experiences. Each of the six items (e.g., “I consider myself as…” and “traditionally, my behavior would be considered as…”) were measured on a 7-point scale ranging from 1 = very masculine to 7 = very feminine (*α* = 0.94).

A measure of gender attitudes (GRA) was also included taken from the wave six World Values Survey 2010–2014 (Inglehart et al., 2014). This is a 5-item measure of attitudes toward traditional gender roles including questions such as “A university education is more important for a boy than for a girl” (*α* = 0.68). Although all five items were measured, one item was removed to improve overall reliability (“being a housewife is just as fulfilling as working for pay”; *α* = 0.74). Answers were measured on a 4-point scale from 1 = strongly agree to 4 = strongly disagree.

### Procedure

Each survey for respective countries was translated and back-translated from English by at least two bilingual speakers of English and Croatian, Dutch, or Thai. Participants were asked to remember their last crying experience that was caused by a negative emotion (e.g., sadness, anger, and frustration) that was not caused by something that they read in a book or watched online, on television, or in a movie and to report when they last cried with a free-text entry to describe it if they wished. Regarding this crying experience, participants were asked what caused their crying, how many people they were with and if they received help (i.e., emotional, informational, and instrumental), and the intensity with which they cried. Participants were then asked how they felt immediately following crying compared to before and their frequency of crying over the last month. They then completed each of the BACS and gender role scales presented in random order and finally answered demographic questions, relating to their age, country of residence, and gender. Participants self-selected to complete the survey based on the memory of their last crying experience and after being screened for relevant age, participants were recruited in their respective countries or online after confirmation of their country of residence. All participants were provided a link to complete this survey online in their own time.

## Results

All analyses were conducted in R version 3.5.2 (R Core Team, 2017). Mediation analyses were tested using *lavaan* (v0.6-3; Rosseel, 2012). The data were first explored to see variations among crying behaviors and evaluations between countries and gender. Overall, women reported crying more frequently, *t*(752) = 11.9, *p* < 0.001, *d* = 0.74, and more intensely than men, *t*(858) = 6.69, *p* < 0.001, *d* = 0.45. Although men and women’s crying frequency and women’s crying intensity did not significantly differ between countries (all *p*s > 0.05), men’s crying intensity showed differences across countries, *F*(22, 355) = 1.65, *p* = 0.033, *η*^2^ = 0.09, with Croatian men showing the lowest crying intensity compared to the other countries sampled (see Table 2). Ratings of change in emotional state following crying showed no difference across men and women; *t*(882) = 0.89, *p* = 0.38, *d* = 0.06. However, beliefs about crying were significantly different between men and women across all three subscales with women believing that crying was more helpful, *t*(769) = 5.24, *p* < 0.001, *d* = 0.36, more unhelpful in social situations, *t*(761) = 2.27, *p* = 0.02, *d* = 0.16, and more unhelpful when alone [*t*(884) = 3.10, *p* = 0.002, *d* = 0.21]. These ratings showed no differences across countries (all *p*s > 0.05). See Table 2 for descriptive statistics.

**Table 2:** Mean (SD) crying behaviors and evaluations separated by country and gender.

Country	Gender	Frequency	Emotion change	Cry intensity	BACS helpful	BACS unhelpful social	BACS unhelpful individual
All countries	Male	1.49 (2.41)	3.41 (1.29)	−0.21 (0.76)	3.09 (1.03)	3.29 (1.19)	2.00 (0.87)
	Female	4.6 (5.12)	3.33 (1.26)	0.16 (0.86)	3.44 (0.94)	3.47 (1.07)	2.18 (0.79)
	Others	4.64 (7.02)	2.86 (1.21)	−0.17 (0.56)	4.29 (0.87)	2.82 (1.04)	1.57 (0.60)
Australia	Male	1.62	3.37	−0.28	2.98	3.33	2.11
	Female	5.74	3.43	0.20	3.13	3.41	2.11
	Others	7.67	3.67	−0.11	3.57	2.92	1.89
Croatia	Male	1.33	3.24	−0.40	3.55	3.13	1.84
	Female	3.89	3.49	−0.04	4.21	3.46	2.09
	Others	1	4	−0.20	5.29	1.5	2
Netherlands	Male	1.01	3.45	−0.06	2.96	3.41	2.04
	Female	3.91	3.57	0.10	3.24	3.73	2.39
	Others	–	–	–	–	–	–
Thailand	Male	1.56	3.23	−0.27	3.06	3.17	1.97
	Female	3.73	2.78	0.11	3.5	3.03	1.84
	Others	2.25	2	−0.24	4.5	3.38	1.17
United Kingdom	Male	1.9	3.75	−0.03	2.92	3.42	2.04
	Female	6.02	3.25	0.41	3.4	3.54	2.29
	Others	4	1	−0.20	5	2.75	1

*Frequency of crying was estimated over the last 30 days; higher scores on emotion change indicate ‘feeling worse’ following crying; higher scores on cry intensity is related to longer crying time with more intense tearing; higher scores on all beliefs scale indicate greater endorsement of the scale*.

### Crying Relationships and Mediation

Due to the combination of ordinal and continuous variables, and non-normal distribution of the crying intensity variable, initial bivariate correlations were conducted using Spearman’s rho with *α* adjusted to <0.005 given multiple comparisons. This analysis found significant relationships between the TMF scale, self-reported gender, crying intensity, and the extent to which crying was believed to be helpful. The GRA scale was also correlated with gender, TMF, and crying intensity but not related to the BACS_Helpful_ scale, see Table 3. However, emotion states following crying only displayed a relationship with helpfulness ratings on the BACS.

**Table 3:** Correlations between self-reported gender, gender roles, beliefs that crying is helpful (BACS_Helpful_), crying intensity, and emotion change following crying.

	TMF	GRA	BACS_Helpful_	Cry intensity	Emotion change
Gender	0.758**	0.311**	0.166**	0.221**	−0.035
TMF	–	0.331**	0.214**	0.185**	−0.040
GRA		–	0.017	0.150**	0.040
BACS_Helpful_			–	0.096*	−0.443**
Cry intensity				–	0.002
Emotion change					–

Spearman’s rho *p < 0.005, **p < 0.001; gender = male (1), female (2); TMF, traditional masculinity/femininity scale; GRA, gender role attitudes.

These relationships indicate that compared to masculine and more traditional gender roles, more feminine and less traditional gender roles was related to greater crying intensity, and beliefs that crying was more helpful overall. Similarly, women were more likely to endorse more feminine and less traditional gender roles, to believe that crying was helpful, and to cry more intensely than men in the sample. Finally, those who believed that crying was helpful were also more likely to feel as though their emotional state improved following crying.

As no relationship was observed between either measure of gender roles and emotional change, a mediation model was only tested with each gender role measure predicting crying intensity and mediated by beliefs that crying is helpful (BACS_Helpful_; see Figure 1). To ensure time since crying was controlled for, this variable was dichotomized following examination of the distribution of crying into recent crying (crying in the last week or earlier, *N* = 335) or non-recent crying (crying more than a week ago, *N* = 559). Because the crying intensity variable displayed a non-normal distribution, bootstrapping was utilized at 1,000 samples. These results revealed significant direct effects of both predictors. However, helpfulness, as rated on the BACS, did not mediate the relationship between either self-ascribed gender roles or attitudes toward gender roles and crying intensity when accounting for the time since crying occurred [*ab* = 0.008, *p* = 0.20, CI (−0.003, 0.023)]. Secondary mediation analyses were conducted to check if these effects were consistent across countries. These analyses also found no mediation across countries. See Table 4 for all mediational results.

**Table 4:** Results overall and across countries testing if the relationship between gender roles and crying intensity is mediated by beliefs that crying is helpful.

	Estimate *ab*	SE	*p*	Lower CI	Upper CI
All countries	0.008	0.006	0.200	−0.003	0.023
Australia	0.009	0.013	0.501	−0.011	0.039
Croatia	0.018	0.020	0.386	−0.014	0.086
Netherlands	0.012	0.012	0.325	−0.005	0.043
Thailand	0.011	0.030	0.721	−0.041	0.079
United Kingdom	0.037	0.032	0.250	−0.018	0.110

*ab, indirect effect; SE, standard error; CI, confidence interval; p, significance*.

#### Mediation Exploration

Given that there was no correlation found between BACS_Helpful_ and the GRA scale, to check if the lack of relationship between these variables influenced the mediation, a second mediation model was analyzed using only the TMF scale. This analysis confirmed the previous results finding that there was no mediation of crying intensity and gender roles measured through the TMF scale *via* BACS_Helpful_ [*ab* = 0.009, *p* = 0.20, CI (−0.005, 0.024)].

To explore the assumption that gender roles are a better predictor of crying behaviors than gender alone, a second mediation model was suggested by a reviewer. This model found partial mediation of the relationship between gender and crying intensity, but only through the GRA scale [*ab* = 0.047, *p* = 0.02 CI (0.011, 0.090)] and not the TMF scale [*ab* = −0.017, *p* = 0.79, CI (−0.150, 0.115)]. These results highlight that gender and attitudes toward gender roles likely both play a role in how intensely people cry, *c*′ = 0.366, *p* < 0.001, CI (0.261, 0.476).

### Social Context Effects

Correlational analyses and pairwise *t*-tests were employed to understand the relationship between emotion following crying and the number of people present when crying, with neither finding significant relationships between variables. Specifically, no correlation was revealed between emotion and the number of people present, *r* = 0.02, *p* = 0.60. Furthermore, when compared against two, three, or more people present when crying, having just one-person present did not appear to influence emotion, all *p*s > 0.05. In fact, further comparisons against people who were alone also found no differences in their ratings of emotion when compared to those with people present, *p*s > 05. No differences were found among these analyses when explored by country.

People were most likely to cry around someone who was known to them (95%), compared to a stranger (5%), and more likely to report receiving help overall (82%), compared to not (18%). A chi-square analysis found a significant relationship between reported help and if the persons in the social context were known, *𝑥*^2^(1, *N* = 421) = 8.00, *p* = 0.005. However, as very few people cried around strangers, an additional analysis examined if the strength of the relationship with those present impacted whether help was perceived to be provided, specifically the presence of “acquaintances” compared to a “friend/family member.” These results revealed that when a person cries in the presence of someone they know, how well they know that person has no influence on whether help was perceived to be provided, *𝑥*^2^(1, *N* = 391) = 0.11, *p* = 0.74. A *t*-test was used to explore whether emotion improved when the person was known to the crier that yielded no significant difference but did show a large effect size *t*(418) = 1.36, *p* = 0.18, *d* = 0.56. As only six people reported crying around strangers compared to 414 people who reported crying around people they knew, this large effect size suggests the relationship would likely exist in a more equally distributed sample.

**Figure 2:**
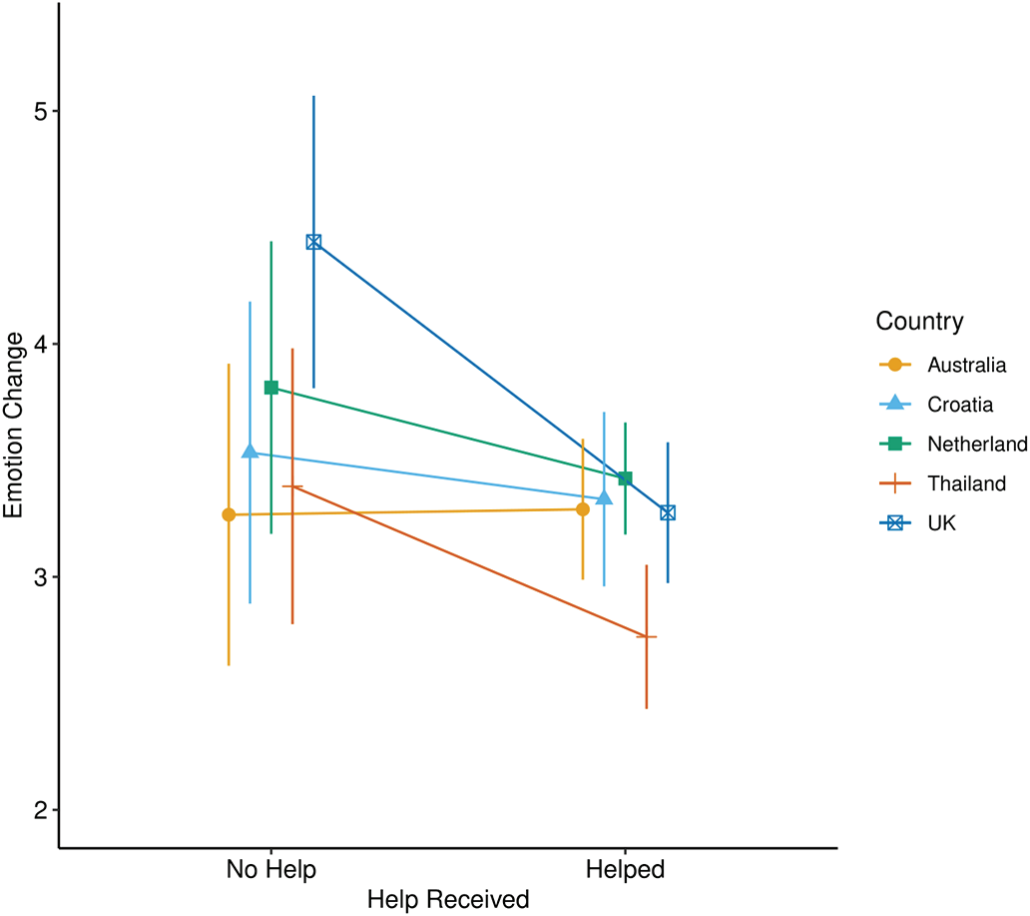
Plot of emotional change ratings whether help was provided when crying in a social context compared by country. Emotion change scores range from 1 = much better to 7 = much worse.

To understand whether emotion improved most when help was perceived to be given, compared to when no help was perceived, a *t*-test was utilized and found a significant difference between the two groups, *t*(436) = 2.84, *p* = 0.005, *d* = 0.35. Further exploration found that for those who were in a social situation, there was no interaction of country and help reported (see Figure 2). However, there was a significant main effect of country on reported emotional change following crying, *F*(4,428) = 3.06, *p* = 0.02, *η*^2^ = 0.03. Further *post hoc* comparisons with Tukey’s corrections revealed that participants living in Thailand rated a better emotional state overall when crying in a social context, compared to those from the United Kingdom, *t*(428) = 3.22, *p* = 0.01, with no other significant differences emerging.

Finally, a correlational analysis was used to understand the relationship between emotional change following crying and beliefs that crying is unhelpful in social contexts. Results showed that worse emotional change ratings following crying were significantly related to stronger beliefs that crying is unhelpful in social contexts, *r* = 0.16, *p* < 0.001. This relationship was found to be significant for both people who were exposed to social contexts, *r* = 0.16, *p* = 0.001, and people who were alone, *r* = 0.15, *p* < 0.001, with a Fisher *r*-to-*z* transformation indicating no significant differences between these groups (*z* = 0.13, *p* = 0.89). When analyzing these correlations across countries, however, it was revealed that one country (i.e., Thailand) was driving this effect with no other countries showing significant relationships either socially or alone with emotional change and beliefs about crying (*r*_*Thai*Social_ = 0.44, *p* < 0.001, *r*_*Thai*Alone_ = 0.26, *p* = 0.008) with a Fisher *r*-to-*z* transformation comparing correlations indicating no differences between the two groups (*z* = 1.28, *p* = 0.20).

## Discussion

The aim of this research was first to understand how gender roles may be involved in crying behaviors and emotional effects, and secondly to gain a better understanding of how the presence of others may impact on participants’ crying experiences. Although this research utilized an international sample, we did not predict cross-cultural differences in the overall relationships between the variables investigated, and largely found none.

### Gender Roles

#### Correlations

We hypothesized that relationships would exist between gender, attitudes toward gender roles, self-ascribed gender roles, beliefs that crying is helpful, crying intensity, and emotional state following crying. This hypothesis was partially supported by our results. In particular, women were more likely to endorse more feminine and less traditional gender roles, with, as predicted, more self-ascribed feminine gender roles related to greater crying intensity and beliefs that crying was more helpful overall compared to people who identified more masculine gender roles. This result reveals, similar to what was found by Ross and Mirowsky (1984) regarding sex-roles, that identification with and attitudes toward gender roles are related to crying behaviors as well as beliefs in the helpfulness of crying. However, we did not find that emotion improvement was related to either self-ascription of gender roles or attitudes toward traditional gender roles. In fact, the only variable that was related to emotion change was with beliefs that crying was helpful. Specifically, those who believed that crying was helpful were also more likely to feel better following crying. Although this relationship appears circular, it helps to understand the widely held public perception that crying is helpful (Cornelius, 2001). We consider that beliefs about crying’s utility may initially influence how a person evaluates their crying experience. However, it is more likely that these processes reinforce one another. That is, a person’s initial evaluation that crying is helpful would likely be adjusted given a crying experience that leaves the crier feeling worse. Testing the direction of this relationship may be possible in future longitudinal research using experiencing sampling, which would allow the measurement of a number of crying experiences and whether there are related changes to beliefs about crying.

#### Mediation

Given a lack of relationship between emotion following crying and gender roles, the second hypothesis was not tested in a mediation model. The third hypothesis, however, was also not supported. That is, when accounting for time passed since crying occurred, beliefs that crying is helpful did not account for the relationship between gender role attitudes and self-ascription and intensity of crying. Similar lack of mediated relationships was found for all countries showing that crying intensity seemed to be accounted for by gender roles. To further understand the relationship between gender roles and crying behaviors, they were explored in a mediation model to understand if crying behaviors might be better explained by a person’s self-perception and attitudes toward gender roles than gender alone (Vingerhoets et al., 2000). These results found that both gender and attitudes toward gender roles meaningfully influenced reports of crying intensity. Specifically, females were more likely to cry more intensely, and the strength of this relationship was partially mediated by females having less traditional attitudes toward gender roles, which in turn predicted more intense crying.

Taken alongside the correlational results, these effects suggest that a person’s gender and the extent to which they subscribe to their gender role (attitudes and self-perception toward gender roles) is highly related to their behavioral crying responses. However, how crying is evaluated appears instead to be related to our attributions of the helpfulness of crying. This may explain why we have previously seen such large differences in crying frequencies between genders but not large differences in how men and women seem to evaluate their crying experiences (Becht and Vingerhoets, 2002; Sharman et al., 2018).

### Crying and Social Help

We also explored the possible social factors involved in crying and emotion change. Contrary to our predictions, we found that ratings of emotion following crying were not affected by the number of people present. Indeed, this effect persisted even when compared with those who were alone and did not differ across countries. This result is not consistent with previous cross-cultural research that found that the presence of one person during a crying episode was related to the greatest emotional improvement (Bylsma et al., 2008). These differences in social responses following crying may have arisen from variation in measurement of emotional improvement, with Byslma et al.’s study measuring change following crying using a 3-point scale, compared to the 7-point scale that was used here.

The results showed some support for our fifth hypothesis, that there is a relationship with whether the crier perceives they received help and whether the crier knows the person who helps them. However, comparatively few people indicated that they cried around strangers or people they did not know. Further analyses found that when crying around friends or family compared to acquaintances, there were no differences in whether help was reported by criers. This suggests that simply knowing someone or a group of people, regardless of how strong that relationship is increases a person’s likelihood to cry around them and that no matter the closeness of the relationship with the crier, known others are perceived to provide the same amount of help. Finally, we also observed a large effect of emotional improvement when crying around someone the person knew. Although this result was not statistically significant, the large effect size suggests this relationship likely exists, but that our samples split between these groups were not of sufficient size to detect an effect, with only six people indicating they cried in the presence of a stranger who were perceived to help. These results do, however, confirm that people are more likely to cry in front of people known to them, rather than strangers (Vingerhoets et al., 1997). Furthermore, these findings support the proposed effects of social crying found in previous research, suggesting that crying encourages others to provide help and succor (Hendriks and Vingerhoets, 2006; Hendriks et al., 2008; Provine et al., 2009). These results also tentatively add to this perspective, indicating that there are distinctions in how help is perceived when provided from strangers or persons known to a crier in whether that help is functional in improving a person’s emotional state.

Support was also found for H6. More precisely, we found that when help was reported by the crier, they tended to rate a better emotional state than those who did not report receiving help from a person present. There were also differences between countries on their reports of emotional change following crying when crying in social situations generally, with participants in the United Kingdom reporting feeling worse following crying compared to those in Thailand. This may suggest a cultural difference among those in the United Kingdom where there is, perhaps, more shame attached to crying in front of others. However, with no other research measuring crying and shame comparing these countries, we can only be tentative in our conclusions here. Overall, it appears that help generally does not alter the course of emotion change following crying in most of the countries studied. Separately, however, some countries did show differentiation in emotion ratings when help was reported, with Thai participants showing the most positive emotion ratings overall when help was reportedly provided. This may mean that there are cross-country differences in how people interpret their emotion states when they feel they have been helped, although we do not know whether this is specifically related to crying situations.

We did not find support for our final hypothesis. The relationship between reported emotional state following crying and the belief that crying is unhelpful in social contexts was the same for those who cried in the presence of others and those who cried on their own. That is, regardless of whether people cried alone or in a social context, people reported worse emotional states following crying when they held beliefs that crying is unhelpful in social contexts. However, this effect appeared to be driven exclusively by Thai participants who tended to rate their emotional state as more negative if they believed crying was unhelpful socially, whether they were crying alone or in a social setting. This effect could be explained by the presence of stronger normative beliefs within Thai society that displaying negative emotions is disrespectful as they can cause distress in others (Cassaniti, 2014). All other countries showed no significant relationships between social beliefs and their emotional state following crying. The results from testing Hypotheses 6 and 7 indicate that, cross-culturally, there are differing social and emotional evaluation characteristics that people are exposed to, which likely affect how they interpret the helping behaviors of others and how that intersects with their evaluations of emotions following crying (Heinrichs et al., 2006; Mesquita et al., 2014).

### Limitations

The cross-country sample of this research provides good generalizability for the results, with sample sizes within and between countries that allow for strong inferences about potential country differences. Nonetheless, there were several limitations to this research. First, this study utilized retrospective self-report, which may have biased participants’ responses. As some participants remembered their last crying experience to be longer than 6 months before, it is possible that participants reported their crying experiences more favorably as time passed (Rottenberg et al., 2008b; Bylsma et al., 2011). Although time since crying was controlled in some analyses, we are still cautious about the conclusions we can make from this research. Furthermore, we note that questions regarding gender that measured global judgments of traits and attitudes may have resulted in greater confirmation to gender stereotypes (Else-Quest et al., 2012). Additionally, greater conformity to stereotypes may have been primed by questions regarding crying, a stereotypical female behavior (Jones and Heesacker, 2012). Perhaps future research would benefit from more accurately measuring gender roles using state-based questions.

Second, the average age of our sample was around 23 years old. Although we included an overall age-bracket between 18 and 40 years, restrictions with sample collections across countries, and attempts to ensure the data were comparable, meant that our samples were much younger. This makes it difficult to generalize our results across age groups, where there may be different influencing social factors, for example, that impact how older or younger adults interpret their crying experience (Blanchard-Fields et al., 2004; Zimmermann and Iwanski, 2014).

Third, it is difficult to derive definite conclusions from our results for those participants who reported that they were “alone” when they cried. As one reviewer noted, there may be a myriad of influencing factors, such as the presence of animals or pets and perhaps memories or imagined social others. Future research would benefit greatly from understanding the potential social role of animals or pets during crying, and even how the imagining of social others may impact emotional evaluations following crying.

### Conclusion

Overall, explorations into crying behavior and emotion change across country and gender found that women cried more frequently and more intensely than men across all countries, yet ratings of emotion following crying were not different between men and women. Our results strongly suggest that these commonly replicated results based on emotion and crying frequency may be explained by gender, prescription to gender roles, and beliefs about whether crying is helpful. That is, self-reported gender and prescription to gender roles appears to be related to the extent to which crying is engaged in, and the intensity of crying (i.e., greater endorsement of femininity relates to more crying and greater crying intensity). Conversely, evaluating whether a crying experience results in feeling better or worse appears instead to be related to individual differences in attributions of the helpfulness of crying, rather than gender.

Beliefs about crying were surprisingly different between genders with women not only showing greater beliefs that crying was generally helpful as was found previously but also beliefs that crying was more unhelpful individually and unhelpful socially, which have formerly shown no differences (Sharman et al., 2018). Although the effects were small, these findings suggest that women may be more sensitive to both positive and negative emotions when crying in different contexts. We do not believe that these results are contradictory but provide further weight to the importance of context specificity. That is, beliefs that crying is helpful are often quite general, such as “crying makes me feel better,” and may be more likely to be influenced by popular opinions (Cornelius, 2001). However, when considering crying in specific contexts such as when alone or in a social space, people may be more likely to draw on specific experiences. Furthermore, as women are more frequent criers, they are more likely to have experienced crying in more varying contexts.

These results also provide some further support for the social function of crying. That is, that crying is a strong social signal that encourages help and succor (Gračanin et al., 2018). Importantly, these results provide a greater understanding of what that helping process means, specifically, that a person is more likely both to cry and to feel that they will receive help around a person that they know, compared to when in the company of a stranger. In addition, that help does not appear to change depending on the extent to which a person is known, with acquaintances and friends/family members perceived to provide equal amounts of support to a crier. Moreover, and notably, when a crier said that they were helped by either instrumental, informational, or physical means, they tended to report a better emotional state following crying than those who did not receive help, but who were around at least one other person. However, it is also important to note that the presence of others has a complex relationship with how people evaluate their emotion following crying, and that the country in which we live may affect how we interpret our crying experience.

## Data Availability Statement

All datasets generated for this study are included in the manuscript/supplementary files.

## Ethics Statement

The studies involving human participants were reviewed and approved by University of Queensland Health and Behavioural Sciences, Low and Negligible Risk Ethics Sub-Committee. The patients/participants provided their written informed consent to participate in this study.

## Author Contributions

The conception and design of the project was completed by LS, EV, and GD. Translations and data collection in each country were completed by LS, EV, and GD for Australia (and some online data collection for the Dutch and UK samples); AV, AF, and KM for the Netherlands; AG and IK for Croatia; HM and SP-a for Thailand; and MB for the United Kingdom. All data were analyzed by LS. The manuscript was drafted and edited by LS with review provided by all co-authors.

### Conflict of Interest

The authors declare that the research was conducted in the absence of any commercial or financial relationships that could be construed as a potential conflict of interest.

## Footnotes

^1^As we were specifically interested in a person’s reports from their last crying experience, we did not include the general measure of crying frequency as a behavioral measure in the predicted mediation model.
